# DNA methylation affects freezing tolerance in winter rapeseed by mediating the expression of genes related to JA and CK pathways

**DOI:** 10.3389/fgene.2022.968494

**Published:** 2022-08-17

**Authors:** Jiaping Wei, Yingzi Shen, Xiaoyun Dong, Yajing Zhu, Junmei Cui, Hui Li, Guoqiang Zheng, Haiyan Tian, Ying Wang, Zigang Liu

**Affiliations:** ^1^ State Key Laboratory of Aridland Crop Science, Lanzhou, China; ^2^ State Key Laboratory of Crop Genetics and Germplasm Enhancement, Nanjing Agricultural University, Nanjing, China; ^3^ College of Agronomy, Gansu Agricultural University, Lanzhou, China; ^4^ Economic Crop Research Institute, Henan Academy of Agricultural Sciences, Zhengzhou, China

**Keywords:** winter rapeseed, freezing stress, DNA methylation, transcriptome, differentially methylated genes

## Abstract

Winter rapeseed is the largest source of edible oil in China and is especially sensitive to low temperature, which causes tremendous agricultural yield reduction and economic losses. It is still unclear how DNA methylation regulates the formation of freezing tolerance in winter rapeseed under freezing stress. Therefore, in this study, the whole-genome DNA methylation map and transcriptome expression profiles of freezing-resistant cultivar NTS57 (NS) under freezing stress were obtained. The genome-wide methylation assay exhibited lower levels of methylation in gene-rich regions. DNA methylation was identified in three genomic sequence contexts including CG, CHG and CHH, of which CG contexts exhibited the highest methylation levels (66.8%), followed by CHG (28.6%) and CHH (9.5%). Higher levels of the methylation were found in upstream 2 k and downstream 2 k of gene regions, whereas lowest levels were in the gene body regions. In addition, 331, 437, and 1720 unique differentially methylated genes (DMGs) were identified in three genomic sequence contexts in 17NS under freezing stress compared to the control. Function enrichment analysis suggested that most of enriched DMGs were involved in plant hormones signal transduction, phenylpropanoid biosynthesis and protein processing pathways. Changes of genes expression in signal transduction pathways for cytokinin (CK) and jasmonic acid (JA) implied their involvement in freezing stress responses. Collectively, these results suggested a critical role of DNA methylation in their transcriptional regulation in winter rapeseed under freezing stress.

## Introduction

Winter rapeseed (*Brassica napus* L.) is a fundamental and nutritious oilseed crops that is widely planted in Northwest China. Freezing stress (<0°C), one of the major abiotic stresses, threatens crop yield as well as quality and causes tremendous agricultural yield penalty and economic losses worldwide (Ding et al., 2020). Moreover, freezing stress can result in membrane lipid peroxidation and cellular structural damage and reduce the activities of reactive oxygen species (ROS)-scavenging enzymes during seedling development (Wei et al., 2021a). Hence, adaptation to different environments, especially to extremely low temperature conditions, plays a pivotal role in the widespread geographical distribution for crops.

DNA methylation is a major epigenetic modification and an essential regulator in the regulation of gene imprinting and gene expression during plant development and stress defense (Seymour and Becker, 2017; Zhang et al., 2018; Huang et al., 2019). In apple, DNA methylation of the *MdMYB10* promoter could regulate *MdMYB10* gene expression and fruit pigmentation during ripening (El-Sharkawy et al., 2015). In rice, DNA methylation of the *OsFIE1* was sensitive to temperature, and regulated seed size under heat stress by controlling early endosperm development (Folsom et al., 2014). Some researches reported the regulation mechanism of cold tolerance responses by DNA methylation in plants (Iwasakiet al., 2019; Guo et al., 2019). In recent years, significant progress has been acquired in knowing the molecular mechanism of freezing resistance in winter rapeseed. Liu et al. (2017) showed that the DNA methylation patterns were changed in rapeseed under freezing treatment by whole genome bisulfite sequencing (WGBS). Wei et al. (2021b) identified some DMGs involved in freezing response in developing rapeseed leaves by integrated transcriptome and methylome analysis. However, these studies were mainly subjected to the screening of a broad range of epigenetically regulated genes, and the evolutionary mechanism of how DNA methylation regulates genes to enhance freezing resistance of winter rapeseed was still poorly understood. Meanwhile, the most researches on DNA methylation modification largely focused on model crops and fruits, such as *Arabidopsis*, rice and tomato (Lang et al., 2017; Guo et al., 2019; Liu et al., 2021).

In the present study, we analyzed the overwintering rate and morphological of winter rapeseed freezing-resistant cultivar NTS57 under freezing stress. The whole-genome DNA methylation map and the transcriptome expression profiles of winter rapeseed under freezing stress were generated. In conjunction with DNA methylation data and transcriptomic data, we identified some DMGs involved in freezing stress, and the metabolic pathways enriched by DMGs were further explored. Furthermore, hundreds of TFs were found to be regulated by DNA methylation, many of which were well studied involved in freezing stress in plants. These findings aimed to obtain insights into the roles of DNA methylation in regulation of gene expression in winter rapeseed under freezing stress.

## Materials and methods

### Plant samples, freezing stress treatments and overwintering rate assessment

One freezing-resistant winter rapeseed cultivar NTS57 (NS, with a more than 85% overwinter survival rate at −26°C), widely planted in Northwest China and used in this study, was provided by Gansu Agricultural University (Wei et al., 2021a). Seedlings of the cultivar NTS57 were grown in plastic pots (5 L) filled with soil from 2015 to 2017. When the potted plants grew to the four leaves stage, they were divided into two groups. The treatment group (Treatment, T2) was transferred to a chamber [−4°C, 60% humidity, 12 h/12 h (day/night) photoperiod, and 350 μmol m^−2^·s^−1^ irradiance] for 24 h, while control group (Control, T0) was maintained in normal condition [22°C, 60% humidity, 12 h/12 h (day/night) photoperiod, and 350 μmol m^−2^·s^−1^ irradiance] for 24 h. The second leaf was sampled from the three control and treated plants, respectively. Each sample contained three biological replicates. Then, the harvest samples were frozen in liquid nitrogen immediately and stored at −80°C for DNA and RNA extraction.

The winter rapeseed seeds were planted in holes in every August from 2015 to 2017, and the overwintering rate was counted after the winter rapeseed turned green in the next year. The overwintering rate is the ratio of the actual seedlings emerging number to the total number of planted seedlings.

### DNA extraction, bisulfite sequencing and data filtering

Total genomic DNA from six samples was extracted using a DNase Plant Mini Kit (Tiangen Biotech, China) following the manufacturer’s protocol. The DNA was sonicated to obtain fragments with a size of approximately 100–300 bp, followed by DNA repair of blunt ends (3′ ends) by the addition of dA and adaptor ligation. Bisulfite modification and conversion of genomic DNA were performed using Methylation-Gold Kit (ZYMO, United States) according to the manufacturer’s instructions. The resulting DNA from freezing-treated and control plants for all samples was subjected to paired-end sequencing using the high-throughput Illumina HiSeqTM 2,500 platform by Gene *Denovo* Biotechnology Co. (Guangzhou, China). High quality clean reads were obtained by removing reads containing more than 10% of unknown nucleotides and more than 40% of low quality bases from raw data.

### Identification of differentially methylated regions

The methylation level was calculated based on methylated cytosine percentage in different regions of the genome. To identify differentially methylated regions (DMRs) between two samples, the minimum read coverage to a methylation status for a base was set to 4.0, a sliding-window approach with 200-bp was used to evaluate DMRs. For each sequence context (CG, CHG and CHH) according to different criteria: for CG, numbers of GC in a window ≥5, absolute value of the difference in methylation ratio ≥0.25, q ≤ 0.05; for CHG, numbers in a window ≥5, absolute value of the difference in methylation ratio ≥0.25, q ≤ 0.05; for CHH, numbers in a window ≥15, absolute value of the difference in methylation ratio ≥0.15, q ≤ 0.05.

### Ribonucleic acid extraction and ribonucleic acid sequencing

Total RNA from six samples containing three biological replicates was ground in liquid nitrogen and extracted using the TRIzol Reagent (Tiangen Biotech, China) according to the manufacturer’s instructions. The library construction and sequencing were performed by Gene *Denovo* Biotechnology Co. (Guangzhou, China) on an Illumina HiSeqTM 2,500 platform. After the Illumina sequencing, three replicates raw sequences for each sample were filtering to generate clean reads for subsequent analysis.

### Filtering of reads and gene expression analysis

Clean data were obtained removing reads containing adapters, reads containing poly-N and low quality reads from raw data. The high-quality paired-end reads from each sample were mapped to rapeseed reference genome by TopHat v2.0.3.12 as illustrated (Kim et al., 2013). The gene expression levels were calculated and normalized as fragments per kilobase per million mapped reads (FPKM), which can be directly used for identifying differentially expression genes (DEGs) in pair-wise comparisons (Trapnell et al., 2010). Genes with a *p*-value < 0.001 and a value of |log_2_FoldChange| ≥2 by the edgeR package (http://www.r-project.org/) were assigned as DEGs, which related to DMRs were defined as DMGs. The sequenced methylome and transcriptome raw data have been deposited to the SRA at NCBI with the accession number of PRJNA685002.

### Quantitative real time-polymerase chain reaction

The expressions of nine candidate freezing-responsive genes were analyzed by qRT-PCR. The qRT-PCR was performed according to protocol of Wei et al. (2021a). All primers were listed in Supplementary Table S1. The relative quantification (2^−ΔΔCt^) of gene expression was evaluated using comparative cycle threshold method, and each sample was replicated for three times.

### Bioinformatics and statistics analysis

The correlation coefficient between three replicates was calculated to evaluate repeatability of the experimental results between samples. Principal component analysis (PCA) was performed to reveal the structure/relationship of samples by R package gmodels (http://www.r-project.org/). Genes were annotated against Gene Ontology (GO) database (http://geneontology.org/) and Kyoto Encyclopedia of Genes and Genomes (KEGG) database (http://www.kegg.jp/) (Binns et al., 2009; Kanehisa et al., 2016). Enrichment analysis were performed based on GO and KEGG databases. The *t*-test was used for analysis of the significant changes in physiological data (SPSS 19.0, United States) with a confidence interval of 99%.

## Results

### Physiological characteristics responses in winter rapeseed under freezing stress

**FIGURE 1:**
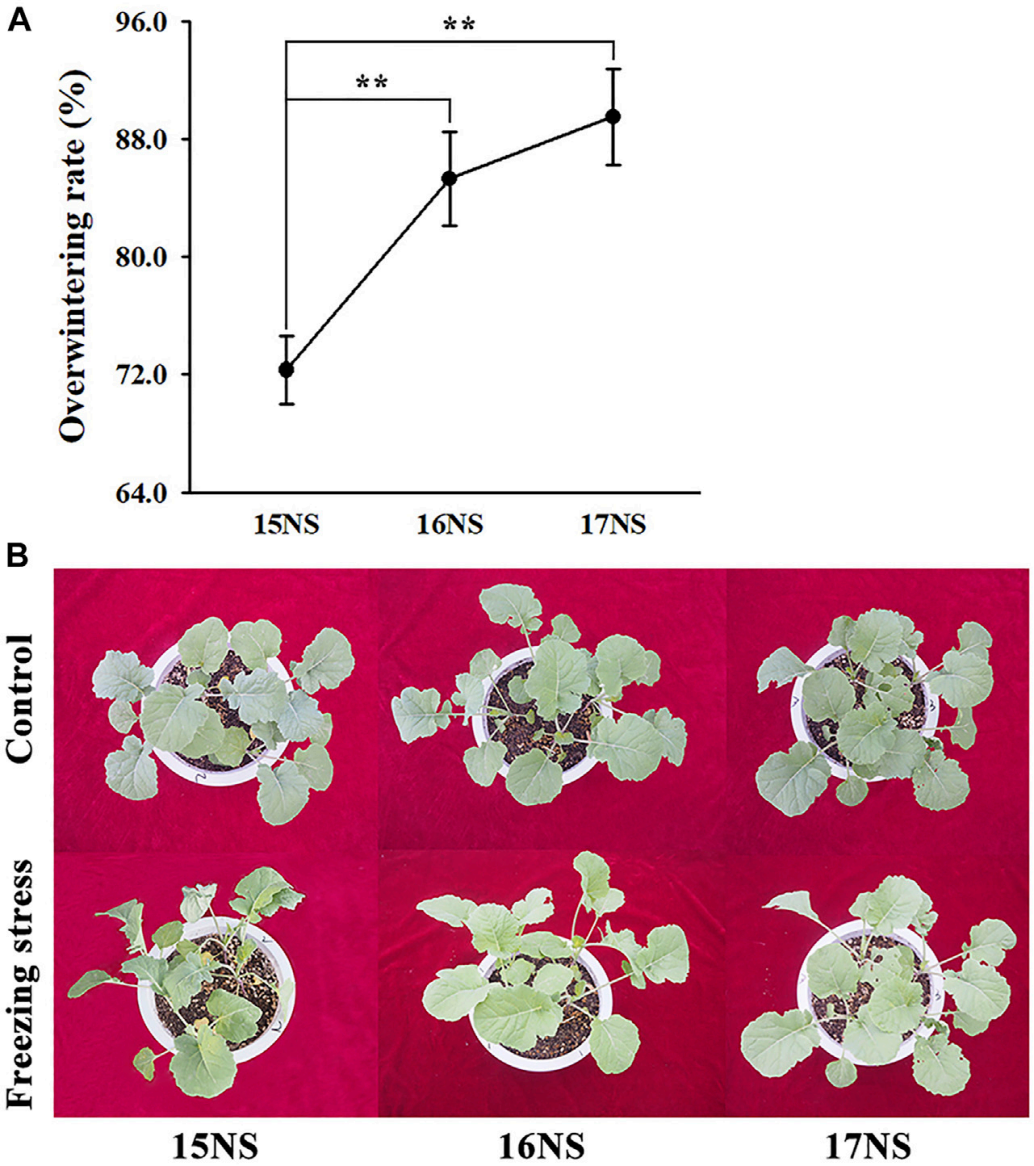
Overwintering rate and morphological changes in winter rapeseed seedling under freezing stress. **(A)**, Overwintering rate of rapeseed NS under freezing treatment. Values are means ± SD from three biological replicates (**, *p* < 0.01); **(B)**, Illustration of 5-week-old winter rapeseed plants under freezing treatment cultivated in 2015, 2016 and 2017.

The freezing stress tolerance of winter rapeseed plants was evaluated. The overwintering rates of winter rapeseed increased year by year, reaching to 72.3, 85.3 and 89.5% in 15, 16 and 17NS plants, respectively. The overwintering rates of 16NS and 17NS plants were significantly higher than that of 15NS plants (Figure 1A). Similar results were observed by morphological. 15NS plants showed more severe damage than 16NS and 17NS plants after 24 h of freezing stress. The leaves of 15NS plants appeared wilting, even the nascent young leaves were found to be died under freezing stress. However, no obvious changes were found in the leaves of 16NS and 17NS plants (Figure 1B). These results suggested that freezing tolerance of NS plants was gradually accumulated.

### Quality control analysis of winter rapeseed methylome and transcriptome

**FIGURE 2:**
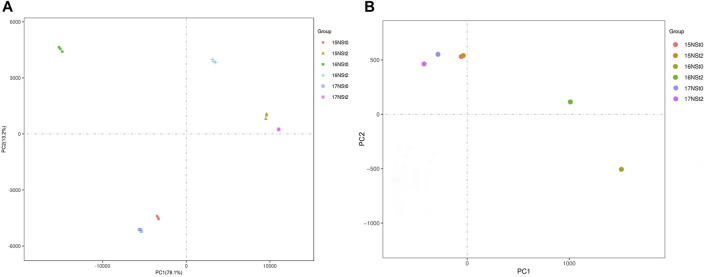
PCA maps of transcriptome and methylome in winter rapeseed NS. **(A)**, PCA of detected genes in RNA sequencing; **(B)**, PCA of detected methylation sites in whole genome bisulfite sequencing.

For the methylome analysis, a total of 167.1–219.9 million sequence reads were generated, over 83.26% of clean reads were uniquely mapped to the rapeseed genome in a single sample, the bisulfite conversion rate for all libraries was close to 99% (Supplementary Table S2). For the transcriptome analysis, a total of 38.53–58.46 million sequence reads were generated, and more than 77.69% of clean reads were uniquely mapped to the rapeseed genome in a single sample (Supplementary Table S2), corresponding to 83,975 genes, including 77,324 known genes and 6,651 new genes in the rapeseed NS (Supplementary Table S3). The PCA indicated that three biological replicates of 15NSt0, 15NSt2, 16NSt0, 16NSt2, 17NSt0 and 17NSt2 had good conformity (Figure 2). In addition, the Pearson’s correlation test exhibited a closely correlation among all samples in methylome (Supplementary Figure S1). A repeatability analysis between three biological replicates of 15NSt0, 15NSt2, 16NSt0, 16NSt2, 17NSt0 and 17NSt2 showed that their correlation coefficient was greater than 0.9 in transcriptome (Supplementary Figure S2). These results indicated a high level of reliability of the methylome and transcriptome analysis.

### Analysis of DNA methylation levels in winter rapeseed under freezing stress

**FIGURE 3:**
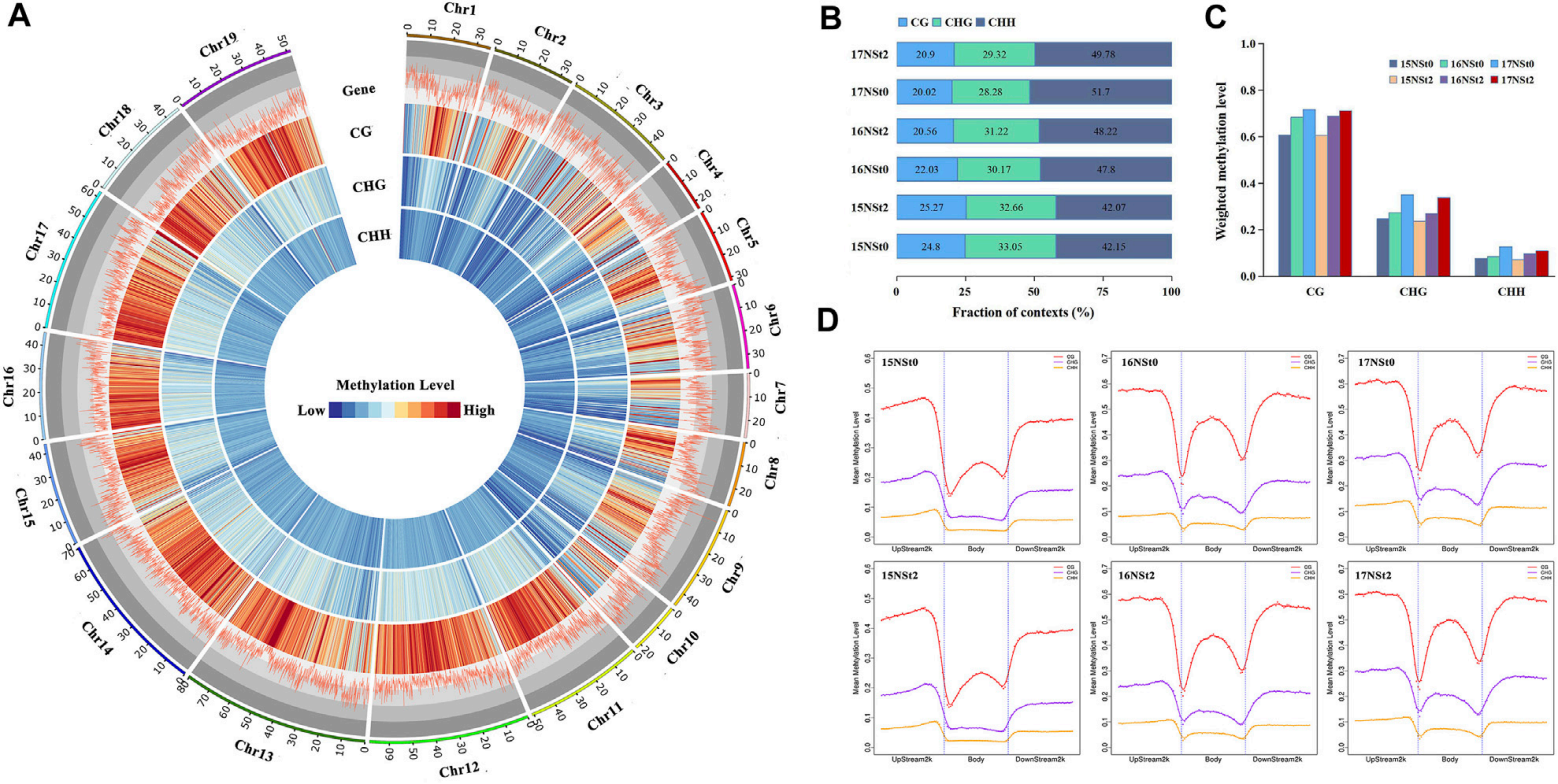
DNA methylation landscape of the winter rapeseed NS. **(A)**, Genome-wide profiles of CG, CHG and CHH DNA methylation across the chromosomes of winter rapeseed: gene density and methylation levels of CG, CHG and CHH sequence contexts in the control and freezing treatment samples, from outer to inner ring. 1-Mb window was used for calculating the gene density or methylation levels; **(B)**, Proportion of CG, CHG and CHH contexts in the total number of methylation sites of winter rapeseed samples under control and freezing treatment; **(C)**, Weighted methylation levels of CG, CHG and CHH contexts of winter rapeseed under control and freezing treatment; **(D)**, Regional methylation levels in three sequence contexts of winter rapeseed across gene-body, upstream 2 k and downstream 2 k regions in samples under control and freezing treatment.

We acquired a single-base-resolution DNA methylation map of winter rapeseed under freezing stress by whole genome bisulfite sequencing technology. The lower levels of methylation were found in gene-rich regions by genome-wide methylation assay (Figure 3A). DNA methylation in winter rapeseed was mainly identified in three genomic sequence contexts, CG, CHG and CHH, which possessed 22.3, 30.8 and 47.0% of the total methylated sites, respectively (Figure 3B). Of these, CG contexts exhibited the highest methylation levels (66.8%), followed by CHG (28.6%) and CHH (9.5%), in complete contrast to their methylation proportions (Figures 3A,C). In addition, the methylation levels at three sequence contexts of winter rapeseed across the gene-body, upstream 2 k and downstream 2 k regions were compared. Our results showed a dramatic decline in DNA methylation around the transcription start site and the transcription termination site, and higher levels in upstream 2 k and downstream 2 k regions, whereas lowest levels in the gene body regions (Figure 3D). These results suggested that the changes in methylation levels in winter rapeseed after freezing stress greatly affect its freezing resistance, and the high methylation level in transcription start site could repress the expression of freezing responsive genes.

### Analysis of differentially methylated genes expression levels in winter rapeseed under freezing stress

**FIGURE 4:**
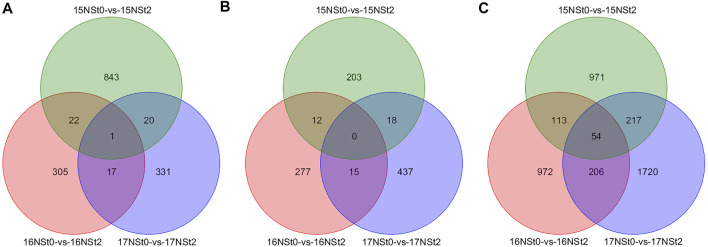
Venn diagram of DMGs identified in three sequence contexts of winter rapeseed 15, 16 and 17NS under freezing stress. **(A)**, DMGs identified in sequence context of CG; **(B)**, DMGs identified in sequence context of CHG; **(C)**, DMGs identified in sequence context of CHH.

All DMRs were analyzed through comparison of NSt2 to NSt0 in 2015, 2016 and 2017, respectively (Supplementary Table S4). In comparison with controls, a total of 1 CG-type, 0 CHG-type and 54 CHH-type shared DMGs were jointly owned in 15NSt0-vs-15NSt2, 16NSt0-vs-16NSt2 and 17NSt0-vs-17NSt2 groups, respectively, among the shared DMGs, 28 were up-regulated and 27 were down-regulated, and the expression patterns of these DMGs in the three groups were completely consistent (Figure 4; Supplementary Table S5). Furthermore, 331 CG-type, 437 CHG-type, and 1720 CHH-type DMGs were identified only in 17NSt0-vs-17NSt2, which were the molecular basis of freezing tolerance in NS at the DNA methylation level (Figure 4). These DMGs were considered as unique genes and used for further analysis.

### Gene Ontology annotation and kyoto encyclopedia of genes and genomes analysis of differentially methylated genes under freezing stress

**FIGURE 5:**
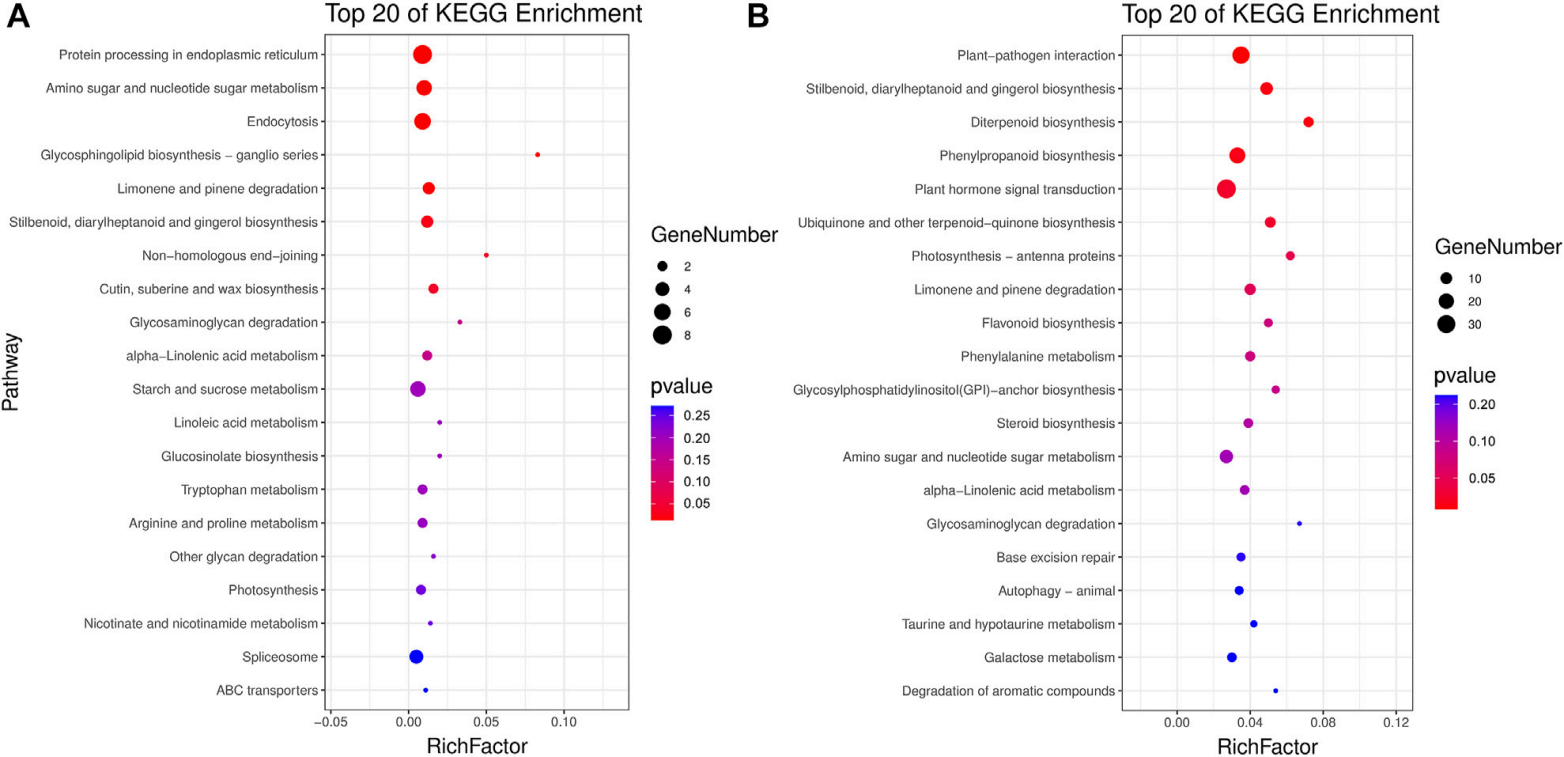
KEGG pathway enrichment. Top 20 KEGG pathways enriched by DMGs identified in sequence contexts of CG **(A)** and CHH **(B)** in winter rapeseed 17NS under freezing treatment.

All unique DMGs identified only in 17NSt0-vs-17NSt2 were subjected to GO classification annotation and KEGG analysis (Supplementary Figure S3; Supplementary Table S6). In total, 57, 66 and 104 metabolic pathways were altered in three genomic contexts containing CG, CHG and CHH, respectively (Supplementary Table S7). Top 20 KEGG enrichment pathways exhibited that plant-pathogen interaction (ko04626), stilbenoid, diarylheptanoid and gingerol biosynthesis (ko00945), plant hormone signal transduction (ko04075), phenylpropanoid biosynthesis (ko00940), diterpenoid biosynthesis (ko00904), and protein processing in endoplasmic reticulum (ko04141) were significantly (*p* < 0.01) enriched by three types of DMGs (Figure 5). These metabolic pathways were believed to be candidate pathways correlated with the freezing tolerance of winter rapeseed.

### Differentially methylated transcription factors under freezing stress

**FIGURE 6:**
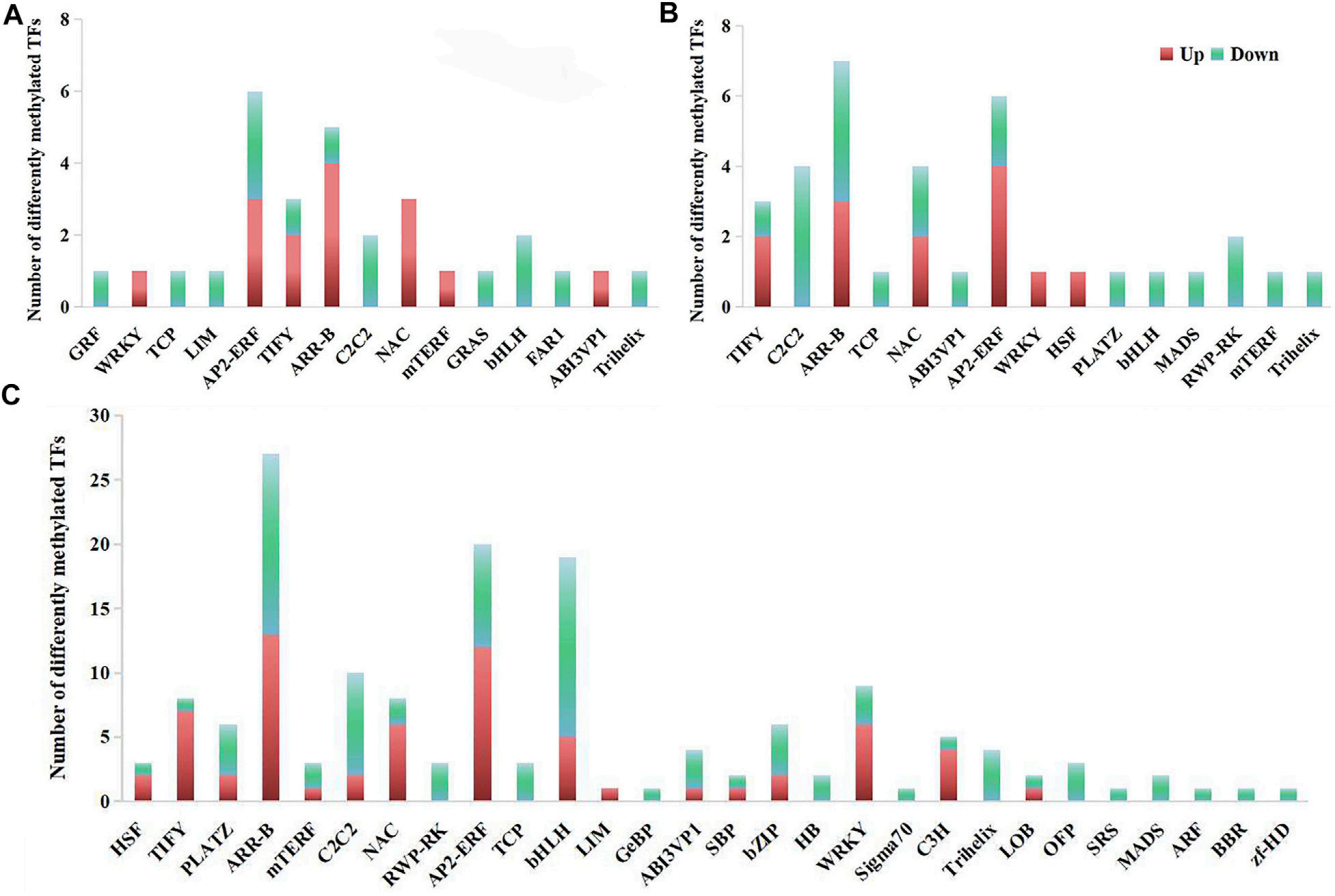
Classification of differentially methylated TFs in winter rapeseed NS. **(A)**, TFs identified in sequence context of CG; **(B)**, TFs identified in sequence context of CHG; **(C)**, TFs identified in sequence context of CHH.

The differentially methylated TFs were screened from differentially methylated genes for further analysis. Totally, 30 CG-type, 35 CHG-type and 157 CHH-type differentially methylated TFs were identified only in 17NSt0-vs-17NSt2 (Figure 6; Supplementary Table S8). Among them, most of the up-regulated differentially methylated TFs belonged to ethylene response factor (AP2/ERF), heat stress transcription factor (HSF), MYB/LUX/GLK transcription factor (ARR-B), NAC domain-containing protein (NAC), WRKY transcription factor (WRKY) and protein TIFY (TIFY), while most of the down-regulated TFs belonged to the transcription factor bHLH (bHLH), zinc finger protein (C2C2) families, MADS-box protein (MADS), NLP9-like protein (RWP-RK), transcription repressor OFP (OFP), transcription factor TCP24-like (TCP), and trihelix transcription factor (Trihelix) families (Figure 6). All results indicated that these differentially methylated TFs were easily activated under freezing stress to regulate the expression of downstream genes response to freezing stress.

### Ribonucleic acid-seq validation by quantitative real time-polymerase chain reaction

**FIGURE 7:**
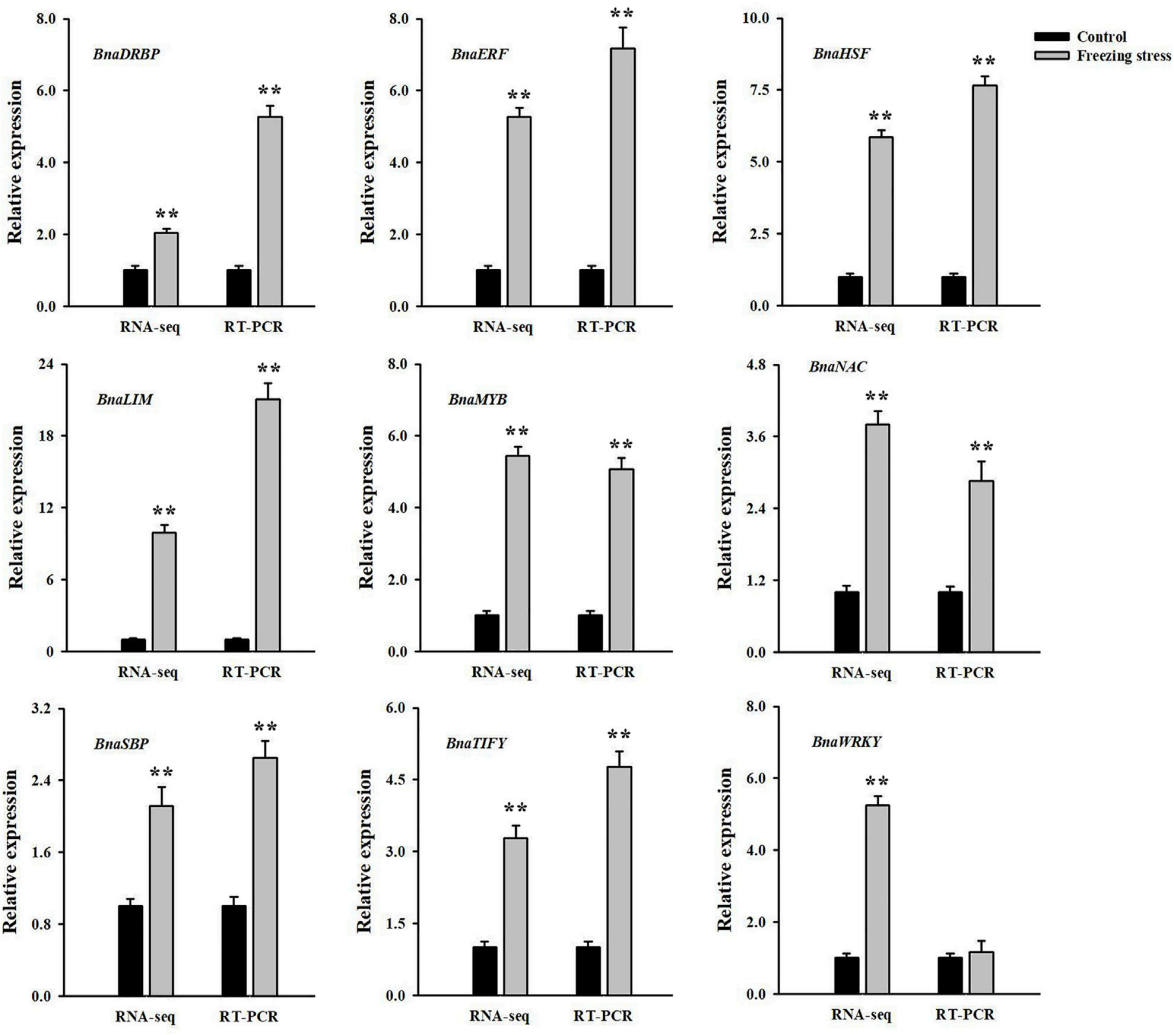
Comparative analysis of mRNA and RNA-seq levels in winter rapeseed NS under freezing stress. Values are means ± SD from three biological replicates in winter rapeseed 17NS under freezing treatment.

The qRT-PCR analysis was used to validate the reliability of our transcriptome. Nine freezing-responsive DMGs involved in signal transduction pathways were selected. Among them, 8 out of 9 were found to be consistent between the mRNA and RNA-Seq levels in rapeseed NS under freezing stress compared to controls. Besides, 1 DMG showed a converse expression pattern at both the mRNA and RNA-Seq levels (Figure 7). These results indicated that the expression patterns detected by qRT-PCR and RNA-Seq were generally consistent, and the result of RNA-Seq was dependable in this study.

## Discussion

It is well known that freezing stress lead to a significant decrease in winter crop yield, especially for winter rapeseed (Guo et al., 2018). It is vital that we discover freezing resistance genes and reveal their regulation mechanisms in winter rapeseed, which will be of great significance for the freezing resistance breeding program for winter rapeseed in the future. In recent years, an increasing number of reports have expounded the effects of freezing stress on winter rapeseed and a number of differentially expressed genes or proteins have been identified by the transcriptome (Pu et al., 2019; Wei et al., 2021a) and proteomics (Xu et al., 2018; Zeng et al., 2018). DNA methylation can regulate plant responses to various stresses (Tong et al., 2021). However, it remains unclear how DNA methylation regulates the freezing tolerance of winter rapeseed. Underlying the role of DNA methylation on freezing stress in winter rapeseed will help to increase the in-depth understanding of the evolutionary mechanism of DMGs in winter rapeseed, which is the primary task to improve winter rapeseed yield and quality. Therefore, in the present study, thousands of DMGs correlated with freezing stress in winter rapeseed were identified and most of them were enriched in KEGG pathways in response to freezing stress. These will be of great importance to elucidate the evolutionary mechanism of DMGs in winter rapeseed under freezing stress.

### The responses of physiological and morphological characteristics in winter rapeseed under freezing stress

Freezing stress can cause a set of physiological and morphological changes, leading to the accumulation of many protective substances in plants (Gao et al., 2019). Freezing stress also results in membrane lipid peroxidation and cellular ultrastructure damage (Deng et al., 2015; Shi et al., 2018), which was comparable with our previous researches (Wei et al., 2021a; Wei et al., 2021b). In this study, under freezing stress, the 15NS plant showed obvious wilting, whereas no significant changes were found in the 16NS and 17NS plants, in compared with controls (Figure 1A), which implied that freezing stress was enhanced year by year. Furthermore, the overwintering rates of 15, 16, and 17NS plants exhibited an increasing trend year by year (Figure 1B), it is entirely in accordance with the phenotypic results. In addition, it has become increasingly evident that ROS scavenging enzymes are indispensable to maintain normal cellular redox homeostasis (Ray et al., 2012). In our previous study, ROS scavenging enzyme activities were obviously increased in NS plants under freezing stress (Wei et al., 2021a). These results are in agreement with findings reported by Zeng et al. (2018).

### The responses of DNA methylation in winter rapeseed under freezing stress

In this study, we generated the single base resolution DNA methylation map of winter rapeseed under freezing stress by WGBS. A total of 22.3, 30.8 and 47.0% of the cytosines in winter rapeseed genome were methylated at CG, CHG and CHH sites, respectively. The average methylation levels of winter rapeseed genome in CG, CHG and CHH sequence contexts were 66.8, 28.6 and 9.5%, respectively, which are much higher than those in rice (Zhang et al., 2015) and Arabidopsis (Niederhuth et al., 2016), lower than those in maize (Xu et al., 2020), cotton (Zhang et al., 2020) and potato (Wang et al., 2018). Taken together, these results suggested that intricate divergence in DNA methylation after abiotic stress treatment is different between species, and there is not a general pattern.

It is generally believed that methylation at promoter regions inhibits transcriptional gene expression, gene region methylation its association with gene transcription is still unclear (Bewick et al., 2016; Lang et al., 2017). In this study, it is interesting to note that most genes displayed consistent methylation patterns between different samples. Higher levels of methylation were found in upstream 2 k and downstream 2 k regions, while the lowest levels were found in gene body regions, in line with previous report by Tong et al. (2021).

### Effects of DNA methylation on the transcription regulation in winter rapeseed under freezing stress

Transcription factors have been reported in plants that monitor the expression of C-repeat binding transcription factor (CBF) by combining relevant cis-elements in their promoters under cold stress. After cold treatment, CBF proteins quickly recognize the promoter regions of downstream cold-regulated genes to activate their expression, thereby enhancing cold tolerance (Zhao et al., 2016). For instance, bHLH transcription factor ICE1, heat shock transcription factor C1, and MYB88/MYB124 transcription factors positively modulate the CBF gene expression that contribute to cold stress (Park et al., 2015; Xie et al., 2018). On the contrary, MYB15 negatively regulate the transcription expression of CBF (Kim et al., 2017). In the present study, most of AP2/ERF, MYB, HSF, NAC, TIFY and WRKY transcription factors were up-regulated in NS, while transcription factors bHLH, C2C2, MADS, OFP, RWP-RK, TCP, and Trihelix were down-regulated (Figure 6), indicating that these differentially methylated TFs might regulate related gene transcription expression in response to freezing stress in winter rapeseed.

Extensive efforts have shown that membrane-located sensors mitogen-activated protein kinases (MAPKs), calcium-dependent protein kinases (CDPKs), calcium-binding proteins (CBPs), and calmodulin-like proteins (CLPs) were vital regulators of cold-stress responses in plant (Kidokoro et al., 2017; Liu et al., 2018; Ding et al., 2019). Our results suggested that a total of 1 MAPK, 6 CBPs, and 3 CLPs were up-regulated and associated with plant pathogen interaction pathway, and their methylation levels in the promoter region were decreased (Supplementary Table S7). These are consistent with what has been reported in previous researches, which suggested that gene promoter region methylation is negatively correlated with related gene expression (Bewick et al., 2016; Guo et al., 2018). Interestingly, we found one down-regulated CDPK with reduced methylation in the promoter region, suggesting that the CDPK promoter region methylation is positively correlated with it is expression. In addition, two up-regulated WRKY with different methylation patterns in the promoter region were found, therefore, we speculated that the plasma membrane-located sensors might perceive the Ca^2+^ signal after freezing treatment, following Ca^2+^ influx to activate their related protein kinases to interact with WRKY, and excite the expression of freezing-responsive genes.

Plant hormones, such as abscisic acid (ABA), cytokinin (CK), ethylene (ETH), auxin (IAA), jasmonic acid (JA), salicylic acid (SA) and brassinosteroid (BR), combine endogenous substances with environmental signals to regulate plant growth development and defense (Huang et al., 2016; Hu et al., 2017; Wu et al., 2019). In the present study, a total of thirty-four DMGs were enriched in plant hormone signal transduction pathway (Figure 5B; Supplementary Table S7), among them, six DMGs with reduced methylation were up-regulated and contributed to JA signaling, encoding TIFY protein and jasmonic acid synthetase, indicating that the freezing tolerance of winter rapeseed can be improved by up-regulating key JA genes through reducing their methylation levels, it is in agreement with those reported by Saha et al. (2016); Thirteen out of seventeen DMGs with different methylation patterns were down-regulated and participated in auxin signaling, encoding auxin-induced protein (AIP), auxin responsive protein (ARP), auxin transporter protein (ATP), GH3 auxin-responsive promoter (GH3) and transport inhibitor response protein (TIRP), consistent with the findings by Shibasaki et al. (2009) in *Arabidopsis* that cold stress inhibited the expression and transport of auxin-responsive related genes; three DMGs with reduced methylation were up-regulated and engaged in ABA signaling, encoding abscisic acid-insensitive protein (AAI), protein phosphatase 2C (PP2C) and serine/threonine-protein kinase SRK2C (SRK), while three DMGs with reduced methylation encoding AAI and abscisic acid receptor (AAR) were down-regulated, thence, we speculate that there may be two different ABA pathways involved in freezing tolerance, and one of them was enhanced. Intriguingly, we found that one down-regulated bHLH28 with reduced methylation, which may be likely to interact with JA-related genes to negatively regulate JA signaling in response to freezing stress (Seo et al., 2011). Furthermore, one DMG with reduced methylation was down-regulated, encoding histidine-containing phosphotransfer protein (HPT), which is a signal transducer and can transfer the phosphate from a sensor kinase to a response regulator in the nucleus (Nongpiur et al., 2012). The response regulators (RR) are usually classified into type A and type B, the type-A RRs are negative regulators in CK signaling, whereas the type-B RRs are positive regulators in CK signaling (Hoang et al., 2021). Fortunately, one two-component response regulator with decreased methylation was found in this study, encoded by *ARR8* gene, which was down-regulated. These results indicated that ARR8 is a type-A response regulator and participates in the negative feedback loop of CK signaling pathway together with HPT to respond to freezing stress. In addition, a further novel finding is that one brassinosteroid-responsive xyloglucan endotransglucosylase/hydrolase was up-regulated, which plays potential roles in plant cell wall remodeling for the increased freezing tolerance (Witasari et al., 2019; Takahashi et al., 2021). Taken together, these results suggested that plant hormones, especially in JA and CK, play crucial roles in response to the freezing stress of winter rapeseed.

Phenylpropanoid compounds assemble a large class of secondary metabolites and are important for against multiple abiotic stresses in plants (Ramakrishna and Ravishankar, 2011; Liu et al., 2015). It has been reported that cold stress induced the expression of structural genes 4-coumarate-CoA ligase (4CL), caffeoyl-CoA O-methyltransferase (CCMT), flavone 3′-O-methyltransferase (OMT) and UDP-glycosyltransferase (UGT), as a consequence, flavonoids and lignin accumulated to facilitate the adaptation to low-temperature environments in plants (Catalá et al., 2011; An et al., 2020). Similar results were obtained in the present study, there were fourteen DMGs with decreased methylation, encoding peroxidase (POD), OMT, UGT and CCMT, which were up-regulated and enriched in phenylpropanoid, stilbenoid, diarylheptanoid and gingerol biosynthesis (Figure 5B; Supplementary Table S7); nevertheless, some down-regulated DMGs with decreased methylation, encoding beta-glucosidase 3-like (BG3) and caffeic acid 3-O-methyltransferase (COMT), were also found in this study. These results suggested that the DNA methylation negatively regulate the expression of genes in association to secondary metabolite synthesis, thereby improving the freezing resistance of winter rapeseed under freezing stress.

It is well established that heat shock proteins (HSPs) considered as the targets of HSFs, are associated with cold stress tolerance in plants (Ré et al., 2016; Andrási et al., 2021). ROS are important signaling molecules and it is accumulation is a characteristic cellular consequence of biotic and abiotic stresses. A large number of reports have illustrated that HSFs can be activated by ROS signals, followed alleviate oxidative damage by promoting ROS-scavenger activities (Kollist et al., 2019; Chen et al., 2020). Consistent with our data, one HSP70 enriched in protein processing in endoplasmic reticulum pathway was up-regulated with increased methylation (Figure 5A; Supplementary Table S7); Furthermore, our previous research indicated that freezing stress resulted in a significant increase in antioxidant enzyme activities in winter rapeseed (Wei et al., 2021a); Apart from that, we found two up-regulated DMGs, which encode RNA polymerase II transcription mediator (PTM), belonging to HSP70 family, which has been demonstrated trigger cold regulated genes under cold stress (Hemsley et al., 2014; Wang et al., 2019); Beyond those, combined with some differentially methylated HSFs (Figure 6), these results implied that HSFs might interact with ROS signals to regulate the expression of HSPs under freezing stress. Additionally, two DMGs with decreased methylation, encoding cell division control protein (CDCP) and protein disulfide isomerase (PDI) were found, and they were up-regulated. These are consistent with what has been found in the previous study (Wei et al., 2021a), who suggested that high level expression of CDCP and PDI were induced in winter rapeseed under freezing stress. These results indicated that protein biosynthesis was enhanced by epigenetic regulation, which were beneficial to contribute to the freezing resistance of winter rapeseed under freezing stress.

In summary, we obtained a single-base-resolution methylation map of winter rapeseed and offer new insights into the roles of DNA methylation on gene expression regulation in winter rapeseed under freezing stress. In addition, we generated the landscape from DNA methylation and gene expression profiles of winter rapeseed under freezing stress. All data and results acquired in the present study will not only provide valuable resources for future researches on gene expression regulation in other freezing treated plants, but will also help to strengthen our understanding for the epigenetic mechanism underlying freezing tolerance in winter rapeseed, which is of great significance for future winter rapeseed genetic improvement with high freezing resistance.

## Data Availability

The datasets presented in this study can be found in online repositories. The names of the repository/repositories and accession number(s) can be found in the article/Supplementary Material.
