# Supplementary figures and images for: DNA methylation affects freezing tolerance in winter rapeseed by mediating the expression of genes related to JA and CK pathways
Source: Front Genet. 2022 Aug 17;13:968494. doi: 10.3389/fgene.2022.968494 (PMC9432081; doi:10.3389/fgene.2022.968494)

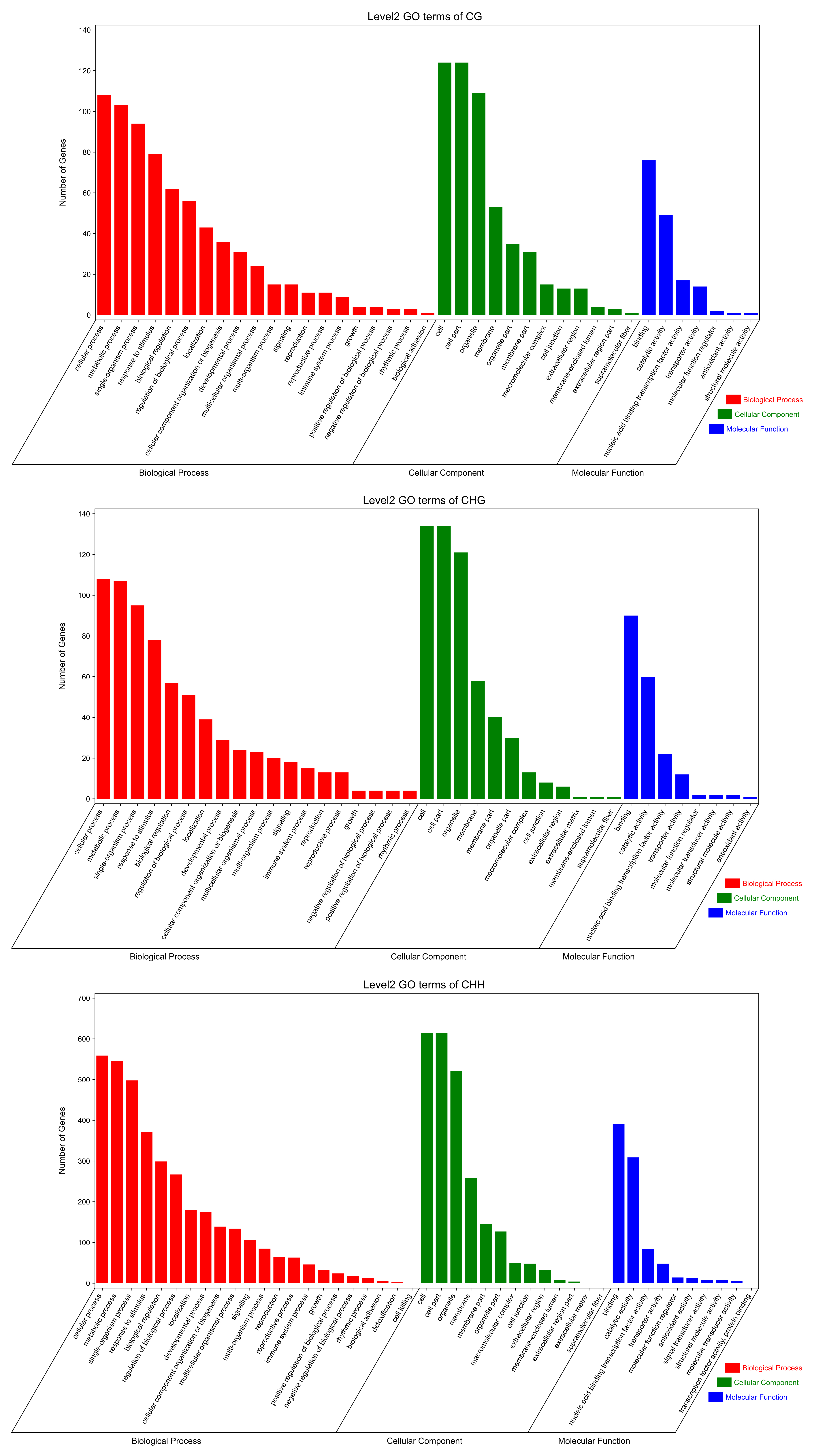

Supplement: Supplementary file 2 [file Image3.JPEG]

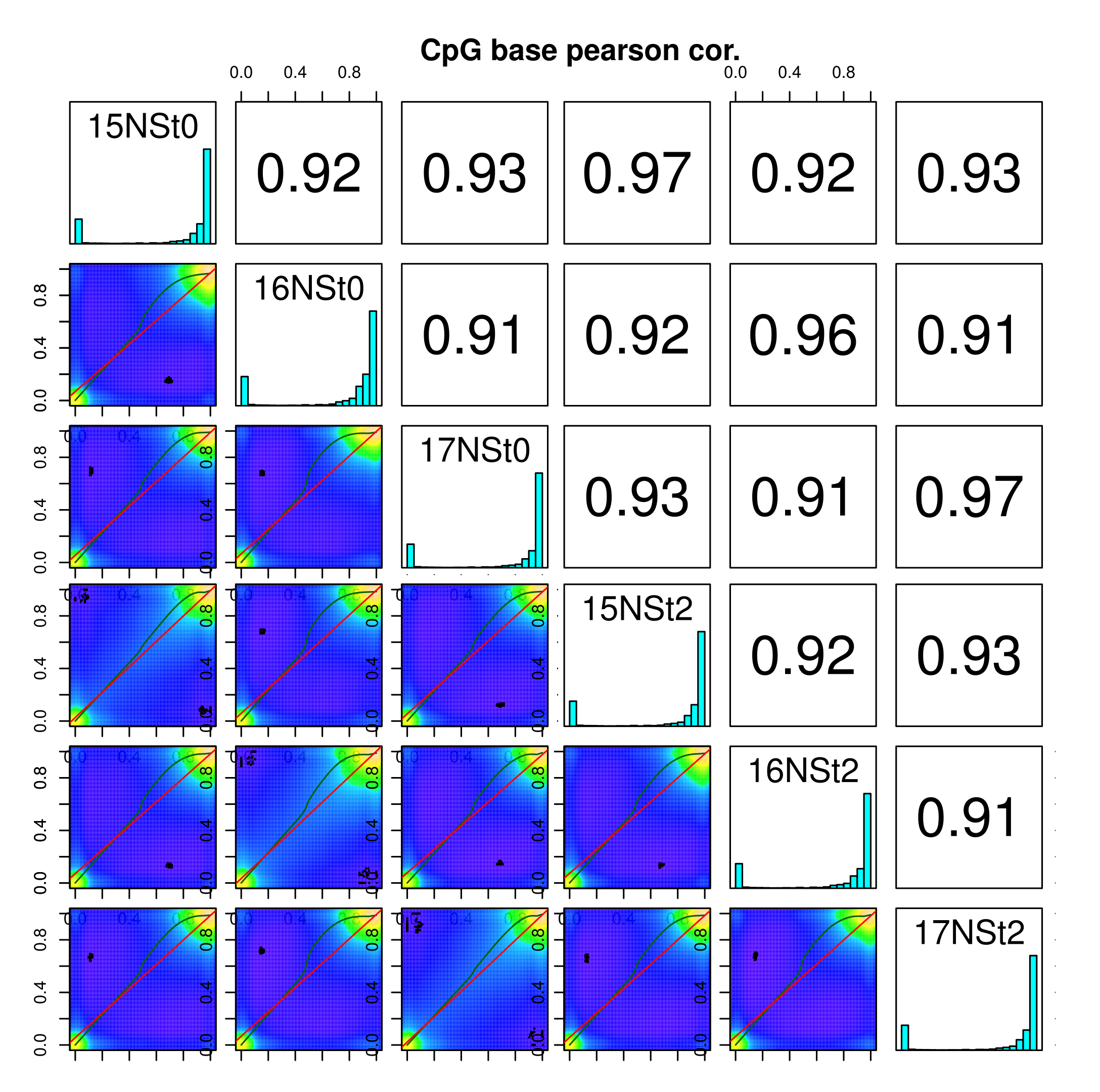

Supplement: Supplementary file 4 [file Image1.JPEG]

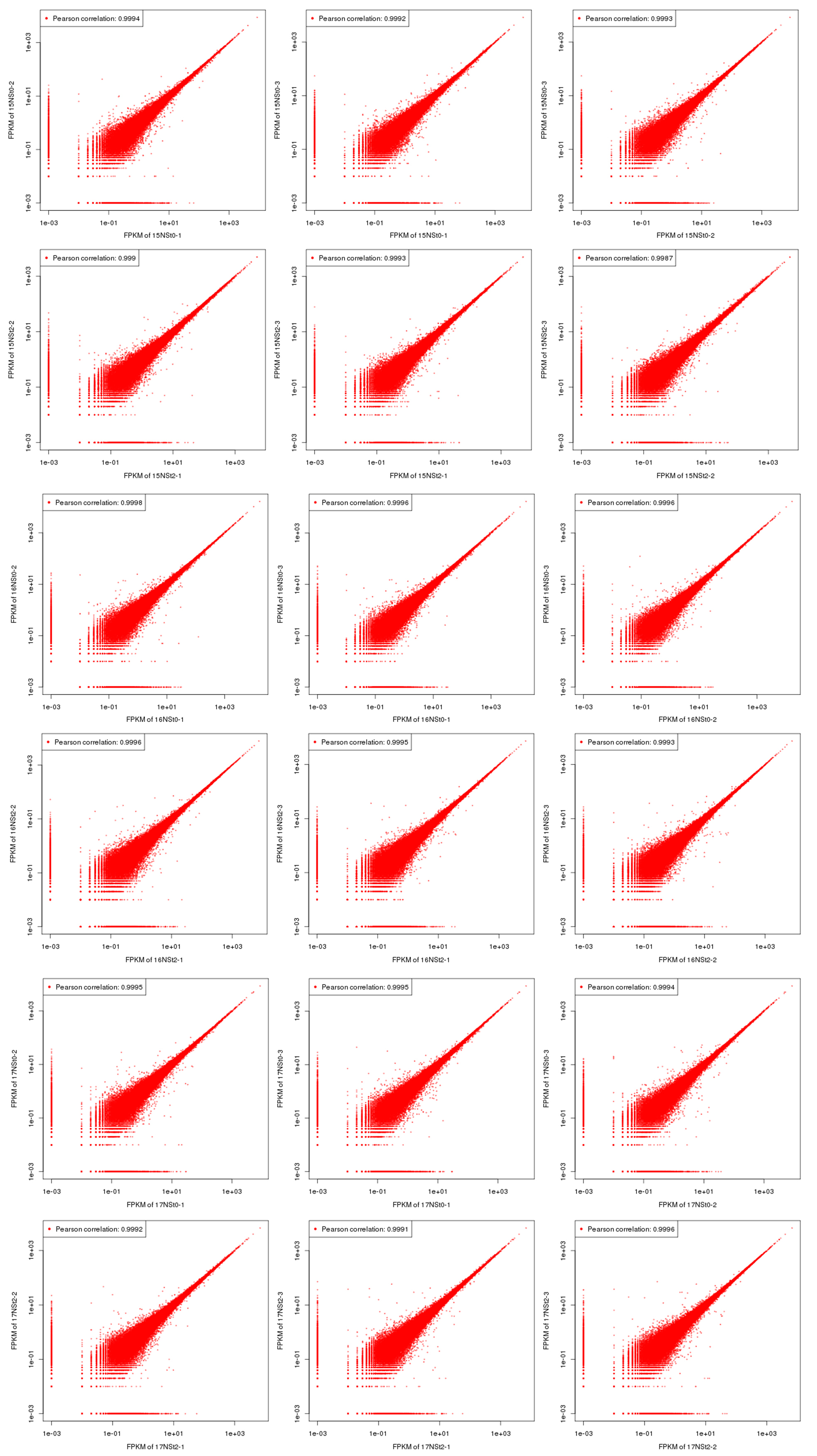

Supplement: Supplementary file 5 [file Image2.JPEG]
